# Examination of the influence of stimuli eccentricity on the inhibitory control in a simon task within virtual reality

**DOI:** 10.1371/journal.pone.0338792

**Published:** 2025-12-30

**Authors:** Dan Bürger, Luisa Peintner, Florian Heilmann, Stefan Pastel, Kerstin Witte

**Affiliations:** 1 Faculty of Humanities, Institute III: Sport Science, Otto-von-Guericke University Magdeburg, Universitätsplatz 2, Magdeburg, Germany; 2 Martin-Luther University Halle-Wittenberg, Universitätsring 4, Halle, Saale, Germany; University of Bologna, ITALY

## Abstract

In certain scenarios, suppressing automated responses and initiating alternative actions is necessary to respond appropriately to stimuli. This process, known as inhibitory control, can be investigated using the Simon task, where stimuli positions are typically task-irrelevant but affect reaction times when stimulus location and response direction differ (Simon effect). Given the importance of reacting to peripheral stimuli in sports and everyday life, this study aimed to examine the influence of stimulus eccentricity on the Simon effect. To this end, a virtual reality version of the Simon task was developed, in which red and green stimuli appeared at different eccentricities (22°, 45°, and 60°), eliciting hand movements to the left or right. Virtual reality allowed for precise control of experimental conditions and simultaneous data collection. Reaction times to congruent (matching stimulus location and response direction) and incongruent (mismatched) stimuli were compared across eccentricities. The results showed significantly larger Simon effects (reaction time differences between incongruent and congruent trials) with increasing stimulus eccentricity, with a greater increase in reaction times for incongruent conditions compared to congruent conditions. As the Simon effect stems from the discrepancy between stimulus location and response direction, it appears to intensify as this discrepancy increases. The study’s limitations include the difficulty of perceiving color stimuli in the visual periphery and its primary laboratory setting, suggesting future research to use geometric shapes instead of colors and incorporate complex virtual reality scenarios to improve the ecological validity of the findings.

## 1. Introduction

The human visual field can be divided into foveal vision (~2–8° eccentricity) and peripheral vision, which can be further subdivided into near-peripheral (8°-30°), mid-peripheral (30–60°), and far-peripheral (> 60°) regions [1,2]. Peripheral vision is crucial in many daily activities (e.g., traffic situations) or various sports (e.g., quickly detecting objects and reacting adequately) [3]. Thereby, reaction times (RTs) to peripheral stimuli are generally longer than those to central stimuli [4], and increase with greater stimuli eccentricities [5].

The adequate response to a stimulus sometimes involves suppression of automated reactions, a cognitive function known as inhibitory control. This plays an important role in many scenarios, for example, in football, when a player intends to pass the ball but realizes that it could be intercepted [6]. One test to quantify the inhibitory control is the Simon task [7], where choice reaction tests are conducted with stimuli appearing at task-irrelevant positions. RTs are prolonged in incongruent trials, whenever the stimulus location (e.g., right side of a screen) contradicts the required response (pressing the left of two buttons); for details, see Hommel [8]. The difference in RTs between congruent and incongruent trials is referred to as the Simon effect [8].

Many models have been proposed to explain the Simon effect and its implications (see [8,9] for reviews). Dual-route models suggest that two competing response pathways give rise to the Simon effect [10,11]. The mismatch between a fast, direct (bottom-up) route, activated by the spatial location of the stimulus, and a slower, indirect (top-down) route that controls for task-specific rules delays the correct response in incompatible trials. The Response Discrimination Account adds that the irrelevant stimulus has to have the potential to discriminate between possible responses to evoke a Simon effect [12]. Other models aim to explain how these conflicting spatial codes emerge. The Attentional-Shift Account suggests that a stimulus is initially detected without processing its spatial characteristics, followed by an attentional shift towards it, forming the spatial code before the non-spatial stimulus feature is interpreted [13]. In contrast, the Referential Coding Account describes a simultaneous processing of all stimulus features, with spatial features derived from the location in the fovea, the absolute position on the display [14], and the location in relation to an alternative stimulus [15]. All these spatial codes interact with the representation of the corresponding response location [8]. The timing of stimulus feature processing is tried to be described by time-difference models, assuming that spatial information is processed more quickly but decays over time, explaining smaller Simon effects with increased RTs [10]. In contrast, the Activation-Suppression Race Model [16] suggests that the location information does not decay passively but is actively inhibited. Thus, longer RTs allow more time for inhibition, reducing the Simon effect. The Common Coding Framework [17] proposes that perceived features (e.g., stimulus location) and action features (e.g., reacting with the left hand) are processed in the same representational medium, linking perception and action. The Theory of Event Coding [18] extends this approach by stating that perceived (e.g., stimuli) and created events (e.g., responses) share common codes. Consequently, the cognitive system treats them equally, leading to activation of a corresponding response, and therefore to faster reactions in compatible Simon task trials.

Most Simon task studies have used stimuli presented in the central visual field (e.g., [19] and [20]). Hommel [21] examined the influence of small eccentricities (0.19°, 3.05°, and 6.10°) on the Simon effect: When eccentricities were randomized, the Simon effect was unaffected, while in the block-wise presentation, the Simon effect was prominent at the smallest eccentricity and diminished with increasing eccentricity [21]. Two studies examined the Simon effect in similar cueing paradigms with targets presented at 8.3° [22] or 29.4° eccentricity [23]. Before the target appeared on one side, a cue was shown either on the target side (cued condition) or the other side (uncued condition). The Simon effect was more prominent in the uncued condition in both studies.

Virtual reality (VR) is a valuable tool for cognitive testing, providing immersive environments with higher ecological validity than traditional laboratory settings [24–26]. Its advantages over screen-based tests have already been demonstrated [27,28], resulting in higher attention levels and a stronger sense of presence, as the real world is entirely excluded [29]. Although cybersickness and acclimatization time must be considered [30,31], the ability to fully control stimuli presentations and simultaneously track head and hand movements makes VR a promising tool for assessing executive functions [25,32]. The Simon task has also been successfully adapted to VR. Qian and colleagues [33] investigated the influence of stimulus 3D depth on the Simon effect, observing no effect of depth. Rocabado and Duñabeitia [34] compared a conventional 2D Simon task with a VR version and found faster reactions in the 2D version, but a similar Simon effect in both conditions. These studies indicate the potential for testing and also training the inhibitory control in realistic scenarios, including peripheral vision. However, first, the Simon effect for greater stimuli eccentricities must be examined.

Although extensively studied, no research known to us has systematically investigated the Simon effect on stimuli presented across the peripheral visual field. Since inhibitory control in response to peripheral stimuli plays a crucial role in many situations, this study aims to examine the Simon effect for peripheral stimuli, using VR to control experimental conditions and simultaneous data collection. Since our study considered much larger eccentricities than others before [21], we hypothesize that the Simon effect will increase with greater eccentricities due to the enhanced incompatibility or compatibility of stimulus location and response direction, induced by a greater shift of attention [13]. Furthermore, in accordance with the Referential Coding Account, when stimuli are presented at random eccentricities, stimuli at greater eccentricities receive a more distinct left or right spatial code, as they are perceived in comparison to stimuli at smaller eccentricities [15].

## 2. Methods

### 2.1. Participants

A priori power calculation using G*Power (version 3.1.9.7) for a 2x3 ANOVA with repeated measures, assuming a moderate effect size, indicated that 28 participants were required to achieve a power of 0.95. To account for potential dropouts or outliers, 30 participants (14 females, 16 males, age: *M* = 24.8 years, *SD* = 3.2 years) were recruited for the study between April 29, 2024 and July 31, 2024. They took part after being informed about the procedure and providing written informed consent. Ethical approval was granted by the first author’s university ethics committee. Exclusion criteria included being under 18 or over 35 years old, having any movement limitations, being red-green colorblind, or requiring glasses during sports. Of the 30 participants, 26 had prior experiences with VR, primarily from participation in other VR studies or through gaming.

### 2.2. Design

At the beginning of the study, participants were informed about the procedure and signed a declaration of consent. Afterward, a demographic questionnaire was completed, followed by two questionnaires administered in a virtual environment using a controller as the input device: one assessing prior VR experience and the Simulator Sickness Questionnaire (SSQ, [35]). Both questionnaires served to acclimatize participants to VR and lasted together approximately five minutes.

Subsequently, the VR tests began, beginning with a reaction test to central stimuli, designed to assess the influence of the following test’s two potential response actions (moving the controller left or right). Afterward, the inhibition task commenced, modeled on the ST, where visual stimuli were presented at different eccentricities relative to a fixation point in the near-peripheral, mid-peripheral, and far-peripheral visual fields.

**Fig 1:**
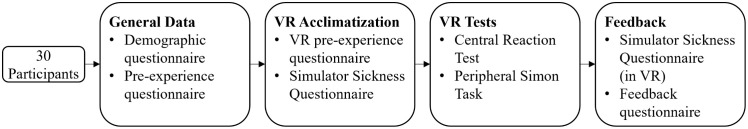
Design of the study.

After the VR tests, the SSQ was completed again in VR to examine potential changes in well-being. Afterward, feedback questionnaires for both VR tests were completed, evaluating the participants’ enjoyment and any potential issues encountered during the VR tests. The design is visualized in Fig 1.

### 2.3. Experimental apparatuses

The virtual environment was presented using the Pimax Vision 5k Super headset (200° diagonal field of view, 2560 x 1440 pixels per eye, 90 Hz refresh rate), connected via cable to a computer (Intel Core i7 CPU, 32 GB RAM, Nvidia RTX 4080 with 16 GB memory). An HTC VIVE Controller (2018) served as the input device, while two SteamVR Base Stations 2.0 tracked the controller and the HMD.

The questionnaires completed in VR were created using the VR Questionnaire Toolkit [36], a Unity package developed to simplify the creation of customized questionnaires in VR. Virtual objects were modeled using Blender (version 3.2.1), and custom C# scripts were developed in Unity (version 2021.3.11f1) to program the behavior of the tests. With SteamVR (version 2.5.4), the virtual environments were transferred into the HMD, and interaction with the controller was enabled. Self-developed MATLAB (version R2022b) scripts were applied to process the recorded data, which were statistically analyzed using SPSS (version 29).

### 2.4. Virtual reality reaction tests

**Fig 2:**
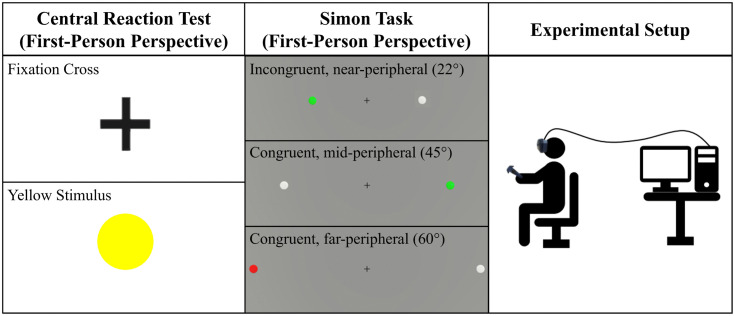
Stimuli of the Central Reaction Test (left) and the Simon task (middle). In the Central Reaction Test, participants reacted to the appearance of the yellow stimulus. In the Simon task, participants reacted to a sphere turning red or green. Congruent conditions appear when a green stimulus is shown on the right side or a red stimulus is shown on the left. All other combinations are considered incongruent. At the beginning of a trial, both spheres were white.

A large background screen covered the entire field of view in both environments. In this study, the HMD served more as a monitor since the environments were not responsive to head movements. The fixation cross remained at the center of the field of view regardless of head orientation, though participants were instructed not to move their heads. All tests were conducted with participants seated on a chair (see Fig 2, right).

#### 2.4.1. Central reaction test.

Participants held the controller in their dominant hand for the Central Reaction Test. The test environment consisted of a white background plane with a black fixation cross. A yellow sphere served as the stimulus, appearing at pseudorandom intervals over the fixation cross (see Fig. 2, left). The test was similar to the S1 task of the Vienna Test System [37], encompassing 28 stimuli. Instead of pressing and releasing a button, participants responded by moving the controller to the left during the first 14 trials or right during the last 14 trials. The controller movement was chosen as input instead of keystrokes to increase ecological validity, as they more closely mirror real-world actions. Participants completed three practice trials for each direction before the test began.

During the Central Reaction Test, the response time, as the time between the stimulus appearance and the controller being moved 10 cm in the correct direction, and the side to which the controller should be moved, were recorded together with the 3D position of the controller at 90 Hz. Incorrect responses were not registered.

#### 2.4.2. Simon task.

**Fig 3:**
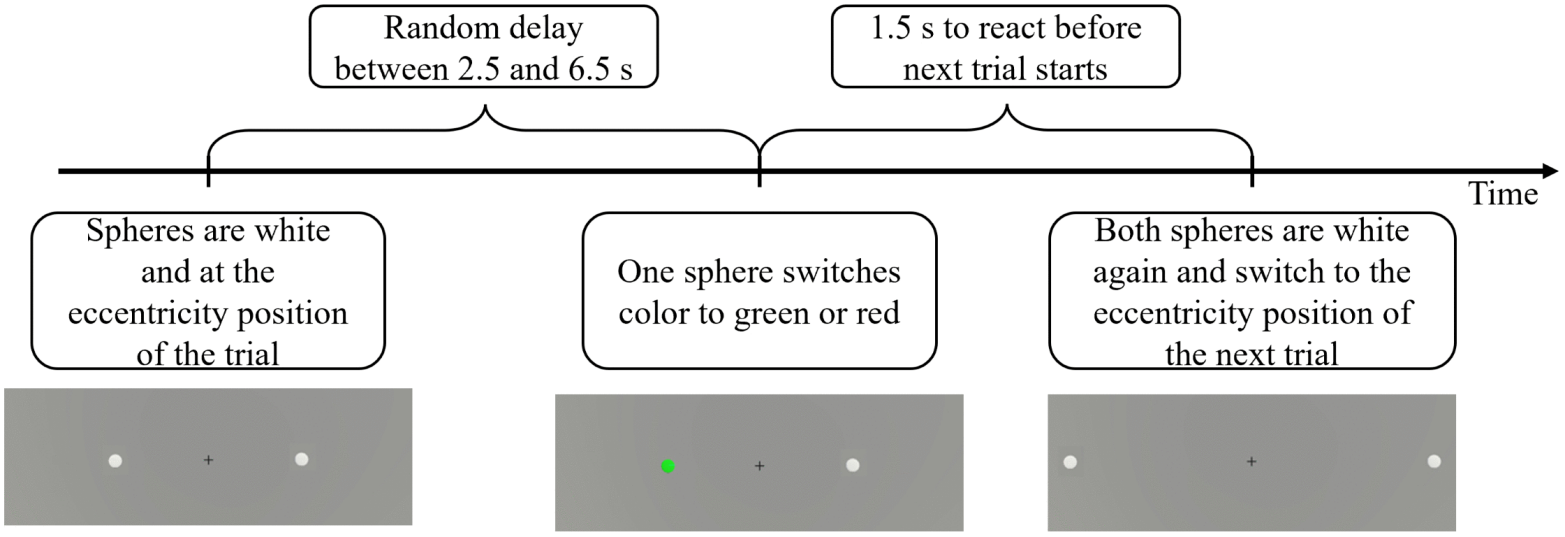
Chronological order of events in the Simon task.

Similar to the Central Reaction Test, the Simon task in VR was completed with the controller held in the participant’s dominant hand. The environment featured a grey background and a fixation cross at the center with two white spheres horizontally aligned, one to the left and one to the right of the fixation cross (see Fig 2, middle). Both spheres were always positioned symmetrically at the same eccentricity on either side, at one of three positions: 22° (near-peripheral), 45° (mid-peripheral), or 60° (far-peripheral) toward each side. The near-peripheral and mid-peripheral numbers of degrees were chosen, as they are approximately in the middle of the regions recommended by Simpson [1]. For the far-peripheral region, 60° was selected, as stimuli in larger eccentricities appeared distorted in the HMD. During each trial, the white spheres changed to one of the eccentricity levels, and after a time interval (between 2.5–6.5 seconds), one of the spheres changed color to either green or red, while the other remained white. Participants then had 1.5 seconds to respond before both spheres turned white again and shifted to the eccentricity level of the subsequent trial. This timing was fixed and did not depend on the participants’ reactions, as illustrated in Fig 3. Participants were instructed to move the controller to the left when a sphere turned red and to the right when a sphere turned green, irrespective of which sphere changed color. The trial conditions, including eccentricity levels, time intervals, and which sphere changed to which color, were pseudorandomized, counterbalanced, and equal for every participant. Congruent trials occurred when the left sphere turned red, or the right sphere turned green, while all other trials were incongruent.

For each combination of eccentricity (near, mid, or far), color (red or green), and condition (congruent or incongruent), participants completed five trials, resulting in 60 trials in total (3 eccentricity levels x 2 sphere colors x 2 conditions x 5 trials). Practice trials were conducted until ten correct reactions were achieved, ensuring task comprehension. The eccentricity levels and selected combinations of sphere color and condition are illustrated in Fig 2 (middle).

During the ST, a timestamp of the Unity system, when a reaction appeared, the response time, as the time between one sphere changing color and the controller being moved 10 cm toward a side, the color of the left and right sphere, and direction of the controller movement, were recorded together with the 3D-position of the controller at 90 Hz.

### 2.5. Data processing

In the Central Reaction Test, the VR system saved a response time for each trial, which is defined as the time between stimulus appearance and the controller being moved 10 cm toward the correct side. To calculate the RT, defined as the time between stimulus appearance and the first movement initiation toward the correct side, excluding the controller’s movement time, a customized MATLAB algorithm was used. The algorithm analyzed the x-coordinate (referring to left and right movement) of the controller’s position within a 0.4-second window prior to reaction detection. The first 1 mm movement was defined as the response initiation and was visually inspected (by plotting the controller position over time and marking the identified time point) for every reaction. The sufficient accuracy of the SteamVR tracking system used has already been demonstrated [38,39].

Outliers in the corrected RTs were identified and excluded for every participant and reactions to the left and right separately using the median absolute deviation (MAD) method [40]. The mean difference between the faster and slower side (overall *M* = 16.7 ms, *SD* = 17.7 ms) was recorded as the ‘central offset’ for every participant individually.

For the ST, a MATLAB script was used to process the data. Since the approximate time points when responses should occur were known, missing reactions were identified by checking for instances where no responses were recorded within a two-second time window around these points. Double reactions were flagged when two responses occurred in quick succession, with less than a one-second difference between them. These occurred when a participant initially reacted to one side but quickly corrected to the other side. Subsequently, errors were determined by searching for trials where participants reacted incorrectly, for example, to the right side when a sphere turned green. All these trials were excluded from the analysis, and the number of errors and double reactions were counted. Since missing reactions mostly occurred when the controller was not moved far enough for the system to register a response, these instances were due to detection failure rather than a true lack of response, making it irrelevant to count them.

Similar to the Central Reaction Test, the movement time was excluded from the response time, using the same MATLAB algorithm, to calculate the RT. All reactions were again visually inspected for accuracy and corrected if needed, and additional double reactions were flagged when the controller initially moved at least 5 cm to the wrong side.

In further processing, the Simon task RTs were individually corrected using the ‘central offset’ to eliminate the influence of movement direction. Afterward, RTs longer than 1.4 s (missed reaction) or shorter than 0.15 s (considered anticipated) were excluded. The remaining trials were categorized by eccentricity (near-, mid-, and far-peripheral) and congruency (congruent and incongruent). Two outliers in the congruent far-peripheral condition were identified and excluded from the analysis. It was ensured that at least five successful trials appeared in each category for each participant. Two participants were excluded due to tracking issues, leaving data from 26 participants for analysis, reducing the power to 0.94. For every participant, mean RTs were calculated for each category and used for the ANOVA.

### 2.6. Statistical analysis

The RTs from the Simon task were analyzed using a 3x2 repeated measures ANOVA, with ‘Eccentricity’ (near-, mid-, and far-peripheral) and ‘Congruency’ (congruent, incongruent) as within-subject factors. Post-hoc tests were Bonferroni-corrected. The assumption of sphericity was met, but normal distribution was violated for all variables (*p* < .05). However, since the ANOVA is robust against this violation, it was computed nevertheless [41]. Furthermore, a one-way ANOVA was conducted for the Simon effect with the within-subject factor ‘Eccentricity’ (near-, mid-, and far-peripheral). Since many outliers appeared in the data set of the errors and double reactions, these were analyzed using Friedman tests with Bonferroni-corrected post-hoc tests.

For the ANOVA *η²*_*p*_ and *η²*_*G*_ were used as effect size (*η²*_*G*__* *_= 0.01 small effect, *η²*_*G*__* *_= 0.06 moderate effect, *η²*_*G*__* *_= 0.14 large effect), as proposed by Lakens [42]. Cohen’s *d* for pairwise comparisons of the ANOVA (*d *= 0.2 small effect, *d* = 0.5 moderate effect, *d* = 0.8 large effect), Kendall’s *W* for the Friedman tests (*W* = 0.1 small effect, *W* = 0.3 moderate effect, *W* = 0.5 large effect), and Pearson’s *r* for the Bonferroni-corrected post-hoc tests (*r* = 0.1 small effect, *r *= 0.3 moderate effect, *r* = 0.5 large effect) [43].

SSQ scores before and after VR exposure were compared for all subcategories separately using Wilcoxon signed-rank tests. No significant changes were observed in the SSQ scores before and after the VR tests (*p* > .05): Nausea (pre-VR: *Mdn* = 0.00, post-VR: *Mdn* = 0.00), Oculomotor (pre-VR: *Mdn* = 18.95, post-VR: *Mdn* = 15.16), Disorientation (pre-VR: *Mdn* = 27.84, post-VR: *Mdn* = 27.84), and Total Score (pre-VR: *Mdn* = 18.70, post-VR: *Mdn* = 18.70).

## 3. Results

### 3.1. Simon effect

**Fig 4:**
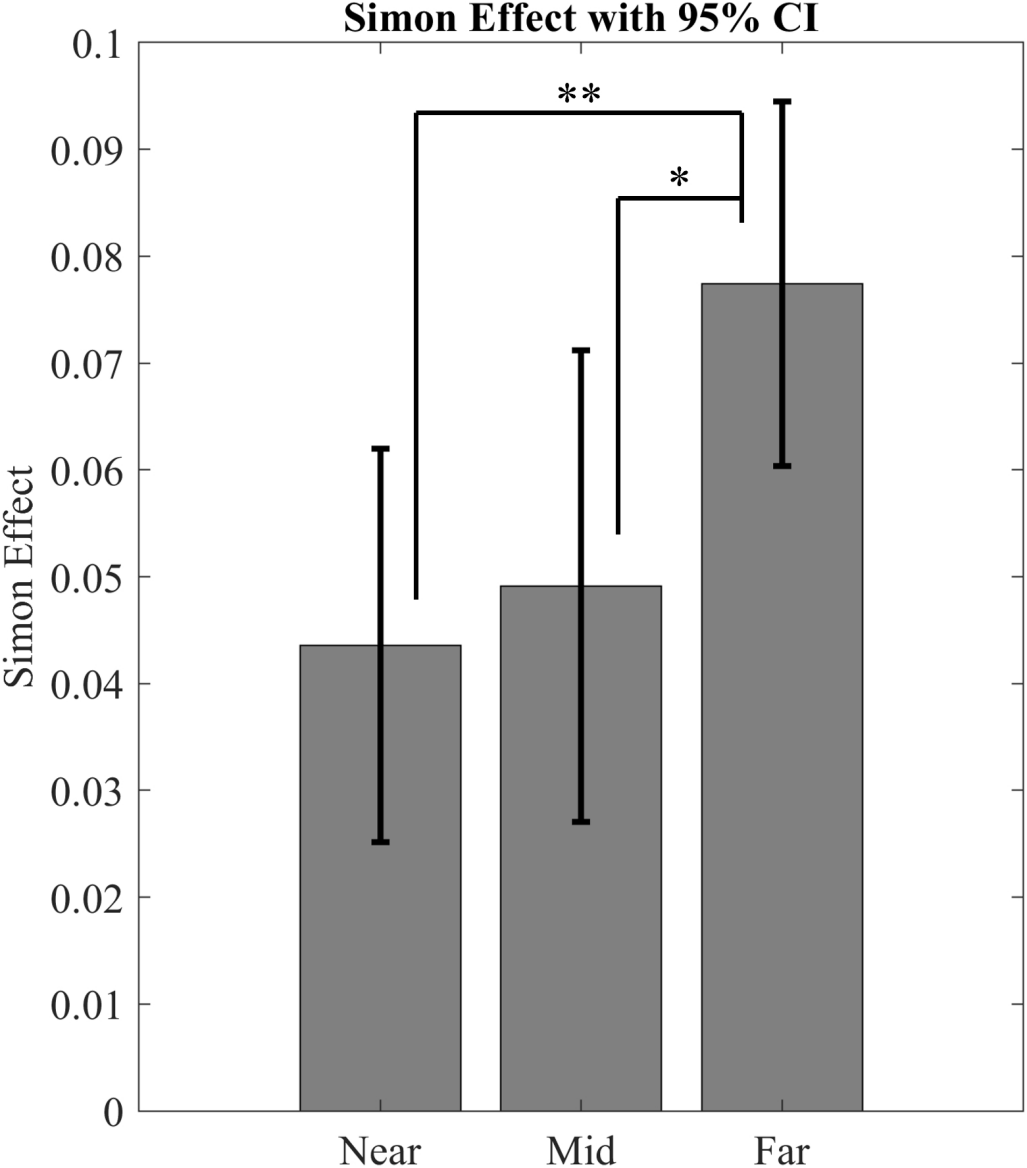
Visualization of the simon effect in the three eccentricity categories.

By subtracting the mean RTs of the congruent trials from those of incongruent trials for each eccentricity category, positive Simon effects result. These indicate faster reactions for congruent trials. The Simon effect differed between the eccentricities (*F*(2,50) = 6.533, *p* = .003, *η²*_*p*_ = .207, *η²*_*G*__* *_= .083 moderate effect), with a greater effect for far-peripheral trials (*M* = 77 ms, *SD* = 44 ms) than for near-peripheral trials (*M* = 44 ms, *SD* = 48 ms, *p* = .008, *d* = 0.657, moderate effect), and for far-peripheral trials compared to mid-peripheral trials (*M* = 49 ms, *SD* = 57 ms, *p* = .038, *d* = 0.527, moderate effect). No significant difference was observed between the Simon effect of near-peripheral and mid-peripheral trials (*p* > .999, *d* = 0.115, no effect). This comparison is illustrated in Fig 4.

Going into more detail, Table 1 shows the mean RTs of congruent and incongruent trials in all three eccentricity categories and their comparison. In all eccentricity categories, significantly shorter RTs were observed in the congruent condition compared to the incongruent condition. Within the congruent condition, only the RTs in the near-peripheral region were significantly shorter than those in the far-peripheral region. In the incongruent condition, the RTs across all eccentricity regions differed significantly, with shorter RTs observed in smaller eccentricities.

**Table 1:** Means and standard deviations of reaction times in the Simon task and statistical comparison between the congruency and eccentricity conditions.

Reaction Times	Near-peripheral	Mid-peripheral	Far-peripheral
	*M (SD)*	*M (SD)*	*M (SD)*
Congruent Trials [ms]	422 (73)	440 (78)	450 (63)
Incongruent Trials [ms]	465 (55)	489 (53)	527 (66)
Factors	**ANOVA**	***p***	**Effect Size**
Eccentricity	*F*(2,50) = 26.639	**<.001**	*η²*_*p*_. = 516, *η²*_*G*_ = .164, large effect
Congruency	*F(*1,25) = 50.768	**<.001**	*η²*_*p*_. = .670, *η²*_*G*_ = .075, moderate effect
Eccentricity * Congruency	*F*(2,50) = 6.533	**.003**	*η²*_*p*_. = .207, *η²*_*G*_ = .013, small effect
Bonferroni-corrected Post-hoc Tests		***p***	**Effect Size** **(Cohen’s *d*)**
Near-peripheral	Congruent vs. Incongruent Trials	**<.001**	0.910, large
Mid-peripheral	Congruent vs. Incongruent Trials	**<.001**	0.857, large
Far-peripheral	Congruent vs. Incongruent Trials	**<.001**	1.744, large
Congruent Trials	Near-peripheral vs. Mid-peripheral	.161	0.397, small
	Near-peripheral vs. Far-peripheral	**.004**	0.708, moderate
	Mid-peripheral vs. Far-peripheral	.652	0.248, small
Incongruent Trials	Near-peripheral vs. Mid-peripheral	**.001**	0.806, large
	Near-peripheral vs. Far-peripheral	**<.001**	1.276, large
	Mid-peripheral vs. Far-peripheral	**<.001**	0.988, large

### 3.2. Errors and double reactions

The numbers of errors and double reactions in each category are presented in Table 2. While for the number of errors, the Friedman test revealed no significant difference across categories (*Χ²*(5) = 10.716, *p* = .057, *W* = .082, no effect, n = 26), it does for the number of double reactions (*Χ²*(5) = 18.676, *p* = .002, *W* = .144, small effect, n = 26). Despite this, the post-hoc tests did not reveal any significant differences (*p* > .623).

**Table 2:** Means and standard deviations of the number of errors and double reactions made in the Simon task separated for each condition.

Number of Errors (max. 10)	Near-peripheral	Mid-peripheral	Far-peripheral
	*M (SD)*	*M (SD)*	*M (SD)*
Simon task congruent	0.19 (0.40)	0.15 (0.37)	0.04 (0.20)
Simon task incongruent	0.27 (0.67)	0.15 (0.46)	0.50 (0.76)
Number of Double Reactions (max. 10)			
Simon task congruent	0.27 (0.60)	0.15 (0.54)	0.08 (0.27)
Simon task incongruent	0.62 (0.94)	0.15 (0.37)	0.54 (0.76)

## 4. Discussion

The present study examined the influence of stimulus eccentricity on the Simon effect in VR. Participants responded to colored spheres at three different eccentricities (near-peripheral, mid-peripheral, and far-peripheral) to the left and right of a fixation cross. Response direction was determined by color, with location (left/right) being task-irrelevant. Results confirmed the presence of a Simon effect in VR, with longer RTs in incongruent trials than in congruent trials, at all eccentricities. Importantly, the Simon effect increases with greater eccentricity, especially in the far-peripheral region. No cybersickness symptoms were found and enjoyment levels were high, with a rating of *M* = 7.3, *SD* = 1.9 on a scale where 0 indicated no enjoyment and 10 indicated high enjoyment, demonstrating that participants were motivated and engaged throughout the experiment.

The size of the Simon effect in this study was larger than in previous studies, where the stimuli were presented in the central visual field [19–21,33,34]. Since the Simon effect in our study increased with increasing stimulus eccentricity, smaller effects in earlier studies align with our hypothesis. In studies that presented the stimuli in the peripheral visual field (8.3° [22] and 29.4° [23]), the effect was comparable to our results. However, a cueing paradigm was used there. In Hommel’s study [21], stimuli were presented at very small eccentricities (< 7°), which are not considered peripheral according to Simpson [1]. Interestingly, unlike in our study, the Simon effect in that study was not influenced by eccentricity. However, the eccentricities were much smaller than those used in ours (22°, 45°, and 60°), raising questions about whether different regions in the visual field affect the Simon effect differently.

Regarding the theoretical perspective, the results can be explained by dual-route models [10,11], where the fast, direct route (activating spatially corresponding response) conflicts with a rule-based, indirect route (color-specific response). The larger Simon effect at greater eccentricities indicates a stronger activation of the spatial code in the far-periphery. Considering the Attention Shift Account, larger attentional shifts were required for stimuli at greater eccentricities before the color could be interpreted. This possibly amplified conflicts in incongruent trials, increasing the time needed for conflict resolution and thus resulting in a greater Simon effect. The Referential Coding Account [14,15] provides an alternative interpretation: For all three eccentricities, the location on the display and the retina should be interpreted as “left” or “right”, but stimuli at greater eccentricities should be seen as more strongly left or right [14]. In addition, an interpretation in relation to the alternative locations is plausible, as, for example, a near-peripheral stimulus on the right side would appear leftward relative to mid- and far-peripheral locations [15]. This could explain why the Simon effect only significantly differed between the far-peripheral location and the other two, as it is the only one that is clearly referenced as left, respectively right. Possibly, the order of eccentricity presentations influences spatial coding, which was attempted to be minimized by including long intervals between trials, as it was not the focus of our study. Together, these models explain the observed increase in the Simon effect with eccentricity.

In contrast, the time-difference models [10] and Activation-Suppression Race Model [16] would expect a smaller Simon effect for larger RTs, which was not observed in our study. RTs increased with eccentricity (eccentricity effect [4]), which should provide more time for location information to decay, respectively, to be actively inhibited. One possible explanation could be that visual acuity decreases in the periphery [44]. This implies that it is more difficult to discriminate between different stimulus features, requiring participants to focus their attention on the stimulus for a longer duration. Consequently, assumingly the location information does not decay as expected, as attention remains directed toward the left or right side. Therefore, the inhibition process of the location information could be prolonged, as cognitive resources are used to interpret the relevant stimulus feature. This interpretation introduces methodical problems, as especially the ability to distinguish colors declines in the peripheral visual field [45]. Since red and green stimuli were used in this study, requiring different responses, this could have influenced the results. However, previous studies have shown that, although being more demanding with increasing eccentricity, red and green stimuli can still be discriminated even at large eccentricities [45,46]. The influence of stimulus discriminability on the Simon effect has already been studied [47–49]. Results largely depended on experimental design. Mostly, when different discriminability conditions were varied in blocks, lower stimulus discriminability resulted in smaller Simon effects [21,47,48,50], where there were no differences when discriminability was randomly varied [47,49]. As in our study, the eccentricities were randomly varied, no difference between different discriminability conditions would be expected. Therefore, the Simon effect in our study should not have been systematically influenced by difficulties in color discrimination at larger eccentricities, as both congruent and incongruent trials would have been affected similarly. Nevertheless, the larger Simon effect observed in the far-peripheral region primarily resulted from an increase in RTs in the incongruent trials. Furthermore, if color discrimination difficulties were a major factor at larger eccentricities, an increase in error rates would be expected in the periphery, which was not observed.

Another potential influence on the Simon effect arises from the participant’s gaze behavior, as unintentional eye movements to verify the stimulus color cannot be entirely excluded. However, saccades are typically performed at a maximum velocity of 700°/s [51], and after a saccade, the eye processes no visual information for ~50 ms [52]. For a 60° saccade, approximately 135 ms would be added to the RTs. Since the RT difference between near- and far-peripheral regions was less than 70 ms, it seems unlikely that eye movements played a significant role.

The eccentricity effect, characterized by increased RTs with greater stimulus eccentricity [4], was observed in congruent and incongruent conditions in our study. The RTs in incongruent trials increased significantly with increasing stimulus eccentricity, showing large effects between all eccentricity regions. In contrast, for congruent trials, only the largest eccentricity difference (near-peripheral vs. far-peripheral) showed significantly increased RTs, with a moderate effect. This disparity likely appears due to the Simon effect, adding to the eccentricity effect in the incongruent condition as eccentricity increased, since this effect also increases with eccentricity. Moreover, some studies have shown that congruent stimuli can lead to shorter RTs than neutral stimuli [53]. Assuming this effect also increases with stimulus eccentricity, the overall eccentricity effect in the congruent condition would be reduced, as observed in our study. However, the absolute RT differences between the eccentricity regions in the congruent condition were comparable to those observed in other studies investigating the eccentricity effect [4,54,55].

In general, the number of errors and double reactions are comparable to those observed in previous studies [19,21,34]. No significant differences were observed between the conditions regarding the number of errors and double reactions, which aligns with [21]. However, in contrast, Borgman and colleagues [19] as well as Rocabado and Duñabeitia [34] observed a significantly greater error rate in incongruent trials, which is also descriptively recognizable in our study. Nevertheless, the overall number of errors (always ≤ 5%) and double reactions (always < 7%) was low, which may have contributed to the lack of significant results. The tendency for more errors in incongruent trials seems logical, as the conflict of stimulus location and response direction only appears in this condition, making it more difficult. In addition, the lack of significant differences in error rates across eccentricities further suggests that the color discrimination in the far-peripheral region was sufficient, as previously mentioned.

## 5. Limitations

While the results of this study are promising, several limitations should be noted. The use of colors to elicit different reactions in the visual periphery poses a challenge. Although colors can be distinguished at larger eccentricities [45,46], processing these stimuli may take more time as colors need to be interpreted. Future studies could employ different geometric shapes and enlarge them in the periphery to minimize this issue [56]. Additionally, the absence of eye tracking prevents us from fully ruling out the use of saccades to verify the stimulus colors. A larger sample size and more trials would also provide greater insight, possibly yielding significant differences in Simon effects across all eccentricity regions. Furthermore, the influence of the order in which the eccentricities are presented could be analyzed. In addition, a more detailed analysis of the number of errors would have been enabled, which, was not the primary focus of this study. Lastly, several advantages of VR were not fully utilized. VR offers higher ecological validity, which is achieved when real-life scenarios are implemented instead of simple stimuli. In our study, VR was beneficial in controlling experimental conditions, as the positions of the head, controller, and stimuli were always known, external distractions were eliminated, and head movements were unnecessary, eliminating the need for a headrest.

## 6. Conclusion

A virtual version of the Simon task was developed, with stimuli appearing at three different eccentricities (22°, 45°, and 60°), providing new insight into how the eccentricity of a stimulus affects the Simon effect. As expected, the difference in RTs between congruent and incongruent trials increased with greater eccentricity, with longer RTs observed for incongruent trials, replicating the Simon effect. Additionally, the eccentricity effect was much more pronounced for incongruent trials, which can be attributed to the Simon effect. At larger eccentricities, stimulus congruency had a more decisive influence on RTs, likely enhancing performance in RTs of congruent trials and, therefore, minimizing the eccentricity effect. In contrast, incongruent stimuli slowed down reactions more significantly at greater eccentricities, amplifying the eccentricity effect and thereby increasing the Simon effect.

The practical implications of these results are diverse. They could advise screen layout optimization in software applications, dashboard designs in cars, or training scenarios in sports, where reactions to peripheral stimuli are crucial, such as for goalkeepers in football. VR should be used to explore the Simon effect in more complex scenarios with different stimuli eccentricities, increasing the ecological validity. On the other hand, it can also be applied to create test and training scenarios for the inhibitory control of reactions to stimuli in the peripheral visual field. In this regard, training effects of these complex scenarios and their transfer to real world situations should be investigated. A possible scenario would be in tennis, where the ball flies toward the athlete, and the opponent’s running direction (left/right) is visualized. No matter on which side of the pitch the opponent is visualized, the athlete should always play the ball against the running direction, creating a Simon task. Importantly, in potential training scenarios, stimuli locations must be task-irrelevant for the decision-making process, as in many sports scenarios, the location is indeed relevant and has to be processed, which should not be impaired by such training.

Future research should examine the influence of eccentricity in smaller steps to better understand the influence of eccentricity on the Simon effect. The integration of eye tracking may be beneficial, as saccades prior to the reactions could be identified, which would have a great impact on the actual eccentricity of the stimulus. Additionally, a greater sample size and an increased number of trials would be beneficial to better explore the influence of eccentricity on the number of errors and the influence of the order of different eccentricities.

## Supporting information

S1 File Analyzed data. This file contains reaction times, Simon effects, number of errors, and number of double reactions used to generate the results.
(XLSX)
